# Supplementary material for: Maternal influenza vaccination during pregnancy and risk of autism spectrum disorder in the offspring
Source: JCPP Adv. 2025 Dec 12;6(3):e70085. doi: 10.1002/jcv2.70085 (PMC13339462; doi:10.1002/jcv2.70085)
Supplement: Supplementary file 1 [file JCV2-6-e70085-s001.docx]

**Maternal influenza vaccination during pregnancy**

**and risk of ASD in the offspring**

**Supporting Information**

**Table S1: A summary of main findings from three large cohort studies examining the association between maternal influenza vaccination during pregnancy and ASD risk in the offspring**

| Study | exposure | Adjusted Risk Estimate (95% CI) |
| --- | --- | --- |
| (Zerbo et al., 2017) | Anytime during pregnancy | 1.10 (1.00 - 1.21) |
|  | 1st trimester: | 1.20 (1.04–1.39) |
|  | 2nd trimester | 1.03 (0.90–1.19) |
|  | 3rd trimester | 1.03 (0.90–1.20) |
| (Ludvigsson et al., 2020) | Anytime during pregnancy | 0.95 (0.81–1.12) |
|  | 1st trimester | 0.92 (0.74–1.16) |
| (Becerra-Culqui, Getahun, Chiu, Sy, & Tseng, 2022) | overall | 1.04 (0.95–1.13) |
|  | 1st trimester | 1.10 (0.98–1.25) |
|  | 2nd trimester | 1.07 (0.96–1.20) |
|  | 3rd trimester | 1.08 (0.94–1.24) |

**Table S2: List of covariates used in the study and their support in the literature.**

| **Covariate** | **Papers** |
| --- | --- |
| Socioeconomic status | (Rai et al., 2012) |
| Maternal age | (Croen, Najjar, Fireman, & Grether, 2007) |
| BMI | (Carter et al., 2023) (Getz, Anderka, Werler, & Jick, 2016) |
| Diabetes | (Carter et al., 2023) |
| Asthma | (Croen, Grether, Yoshida, Odouli, & Van de Water, 2005) |
| Smoking | (von Ehrenstein, Cui, Yan, Aralis, & Ritz, 2021) |
| Inflammatory bowel disease | (Sadik et al., 2022) |
| Antibiotic use | (Hamad, Alessi-Severini, Mahmud, Brownell, & Kuo, 2019) |
| Diabetes/Maternal Diabetes | (Carter et al., 2023) |
| Pre-term birth | (Crump, Sundquist, & Sundquist, 2021) |
| Delivery type | (Huberman Samuel et al., 2019) |
| Birth weight | (Lampi et al., 2012) |
| Gestational age at delivery | (Lampi et al., 2012) |
| Decreased fetal movements | (Zamstein, Wainstock, & Sheiner, 2019) |
| Polyhydramnios, Oligohydramnios | (Chien et al., 2019) |

**Table S3.** **Hazard ratios of ASD associated with influenza vaccine administration during pregnancy without pregnancies occurring between January 1^st^ and December 31^st^ of 2020**

|  | Total number of pregnancies | ASD cases (%) | Unadjusted hazard ratio (95%CI) | Model I ^a^ (95%CI) | Model II ^b^ (95%CI) |
| --- | --- | --- | --- | --- | --- |
| Unexposed during pregnancy | 108,107 | 2,469 (2.3) | - | - | - |
| Vaccination during pregnancy | 37,829 | 1,063 (2.8) | 1.22 (1.14-1.31) | 1.02 (0.95-1.1) | 0.98 (0.90-1.05) |
| Vaccination during first trimester | 10,464 | 313 (3.0) | 1.34 (1.19-1.51) | 1.09 (0.97-1.22) | 1.03 (0.91-1.16) |
| Vaccination during second trimester | 15,233 | 414 (2.7) | 1.16 (1.05-1.30) | 0.97 (0.87-1.08) | 0.93 (0.84-1.04) |
| Vaccination during third trimester | 12,132 | 336 (0.28) | 1.2 (1.07-1.34) | 1.03 (0.92-1.15) | 0.98 (0.88-1.11) |

^a^Adjusted for maternal age at delivery, district of residence, ethnicity.

^b^Adjusted for maternal age at delivery, maternal BMI, gravidity, smoking status, ethnicity, district of residence, maternal comorbidity (asthma, diabetes mellitus), gestational diabetes, oligohydramnios, pregnancy with history of pre-term birth, delivery type, gestational age at birth, infant gender, birth weight

**Table S4: Influenza vaccination and ASD risk in Bedouin Arab Women Living in the South of Israel vs. All Other Women in the Study**

| aHR (95%CI)^a^ | ASD cases (%) | Total number of women | Exposure status | Group |
| --- | --- | --- | --- | --- |
| - | 187 (0.6%) | 31,496 | Unexposed | Bedouin Arab women living in southern Israel |
| 1.23 (0.82-1.83) | 30 (0.8%) | 3,715 | Exposed |  |
| - | 2,391 (2.9%) | 82,464 | Unexposed | Other women in the study  (non-Bedouin) |
| 0.96 (0.90-1.04) | 1,061 (3.0%) | 35,646 | Exposed |  |

^a^Adjusted for maternal age at delivery, maternal BMI, gravidity, smoking status, district of residence, maternal comorbidity (asthma, diabetes mellitus), gestational diabetes, oligohydramnios, pregnancy with history of pre-term birth, delivery type, gestational age at birth, infant gender, and birth weight

**References**

Becerra-Culqui, T. A., Getahun, D., Chiu, V., Sy, L. S., & Tseng, H. F. (2022). Prenatal Influenza Vaccination or Influenza Infection and Autism Spectrum Disorder in Offspring. *Clinical Infectious Diseases*, *75*(7), 1140–1148. Oxford University Press. Retrieved June 18, 2025, from https://pubmed.ncbi.nlm.nih.gov/35174388/

Carter, S. A., Lin, J. C., Chow, T., Yu, X., Rahman, M. M., Martinez, M. P., Feldman, K., et al. (2023). Maternal obesity, diabetes, preeclampsia, and asthma during pregnancy and likelihood of autism spectrum disorder with gastrointestinal disturbances in offspring. *Autism : the international journal of research and practice*, *27*(4), 916–926.

Chien, Y.-L., Chou, M.-C., Chou, W.-J., Wu, Y.-Y., Tsai, W.-C., Chiu, Y.-N., & Gau, S. S.-F. (2019). Prenatal and perinatal risk factors and the clinical implications on autism spectrum disorder. *Autism : the international journal of research and practice*, *23*(3), 783–791.

Croen, L. A., Grether, J. K., Yoshida, C. K., Odouli, R., & Van de Water, J. (2005). Maternal Autoimmune Diseases, Asthma and Allergies, and Childhood Autism Spectrum Disorders. *Archives of Pediatrics & Adolescent Medicine*, *159*(2).

Croen, L. A., Najjar, D. V, Fireman, B., & Grether, J. K. (2007). Maternal and paternal age and risk of autism spectrum disorders. *Archives of pediatrics & adolescent medicine*, *161*(4), 334–40.

Crump, C., Sundquist, J., & Sundquist, K. (2021). Preterm or Early Term Birth and Risk of Autism. *Pediatrics*, *148*(3).

von Ehrenstein, O. S., Cui, X., Yan, Q., Aralis, H., & Ritz, B. (2021). Maternal Prenatal Smoking and Autism Spectrum Disorder in Offspring: A California Statewide Cohort and Sibling Study. *American journal of epidemiology*, *190*(5), 728–737.

Getz, K. D., Anderka, M. T., Werler, M. M., & Jick, S. S. (2016). Maternal Pre-pregnancy Body Mass Index and Autism Spectrum Disorder among Offspring: A Population-Based Case-Control Study. *Paediatric and perinatal epidemiology*, *30*(5), 479–87.

Hamad, A. F., Alessi-Severini, S., Mahmud, S. M., Brownell, M., & Kuo, I. F. (2019). Prenatal antibiotics exposure and the risk of autism spectrum disorders: A population-based cohort study. *PloS one*, *14*(8), e0221921.

Huberman Samuel, M., Meiri, G., Dinstein, I., Flusser, H., Michaelovski, A., Bashiri, A., & Menashe, I. (2019). Exposure to General Anesthesia May Contribute to the Association between Cesarean Delivery and Autism Spectrum Disorder. *J Autism Dev Disord*. Public Health Department, Ben-Gurion University of the Negev, Beer Sheva, Israel. Pre-School Psychiatry Unit, Soroka University Medical Center, Beer Sheva, Israel. Psychology Department, Ben-Gurion University of the Negev, Beer Sheva, Israel. Zlotowski Ce: Springer New York LLC. Retrieved from internal-pdf://144.113.156.76/HubermanSamuel2019_Article_ExposureToGeneralAn.pdf

Lampi, K. M., Lehtonen, L., Tran, P. L., Suominen, A., Lehti, V., Banerjee, P. N., Gissler, M., et al. (2012). Risk of autism spectrum disorders in low birth weight and small for gestational age infants. *The Journal of pediatrics*, *161*(5), 830–6.

Ludvigsson, J. F., Winell, H., Sandin, S., Cnattingius, S., Stephansson, O., & Pasternak, B. (2020). Maternal Influenza A(H1N1) Immunization During Pregnancy and Risk for Autism Spectrum Disorder in Offspring. *Annals of Internal Medicine*, *173*(8), 597–604.

Rai, D., Lewis, G., Lundberg, M., Araya, R., Svensson, A., Dalman, C., Carpenter, P., et al. (2012). Parental socioeconomic status and risk of offspring autism spectrum disorders in a Swedish population-based study. *Journal of the American Academy of Child and Adolescent Psychiatry*, *51*(5), 467-476.e6.

Sadik, A., Dardani, C., Pagoni, P., Havdahl, A., Stergiakouli, E., iPSYCH Autism Spectrum Disorder Working Group, Khandaker, G. M., et al. (2022). Parental inflammatory bowel disease and autism in children. *Nature medicine*, *28*(7), 1406–1411.

Zamstein, O., Wainstock, T., & Sheiner, E. (2019). Decreased fetal movements: Perinatal and long-term neurological outcomes. *European journal of obstetrics, gynecology, and reproductive biology*, *241*, 1–5.

Zerbo, O., Qian, Y., Yoshida, C., Fireman, B. H., Klein, N. P., & Croen, L. A. (2017). Association Between Influenza Infection and Vaccination During Pregnancy and Risk of Autism Spectrum Disorder. *JAMA Pediatrics*, *171*(1), e163609.
